# Paths of Heritable Mitochondrial DNA Mutation and Heteroplasmy in Reference and *gas-1* Strains of *Caenorhabditis elegans*

**DOI:** 10.3389/fgene.2016.00051

**Published:** 2016-04-13

**Authors:** Riana I. Wernick, Suzanne Estes, Dana K. Howe, Dee R. Denver

**Affiliations:** ^1^Department of Integrative Biology, Oregon State UniversityCorvallis, OR, USA; ^2^Department of Biology, Portland State UniversityPortland, OR, USA

**Keywords:** heteroplasmy, mutation, mitochondrial DNA, *Caenorhabditis elegans*, experimental evolution

## Abstract

Heteroplasmy—the presence of more than one mitochondrial DNA (mtDNA) sequence type in a cell, tissue, or individual—impacts human mitochondrial disease and numerous aging-related syndromes. Understanding the trans-generational dynamics of mtDNA is critical to understanding the underlying mechanisms of mitochondrial disease and evolution. We investigated mtDNA mutation and heteroplasmy using a set of wild-type (N2 strain) and mitochondrial electron transport chain (ETC) mutant (*gas-1*) mutant *Caenorhabditis elegans* mutation-accumulation (MA) lines. The N2 MA lines, derived from a previous experiment, were bottlenecked for 250 generations. The *gas-1* MA lines were created for this study, and bottlenecked in the laboratory for up to 50 generations. We applied Illumina-MiSeq DNA sequencing to L1 larvae from five *gas-1* MA lines and five N2 MA lines to detect and characterize mtDNA mutation and heteroplasmic inheritance patterns evolving under extreme drift. mtDNA copy number increased in both sets of MA lines: three-fold on average among the *gas-1* MA lines and five-fold on average among N2 MA lines. Eight heteroplasmic single base substitution polymorphisms were detected in the *gas-1* MA lines; only one was observed in the N2 MA lines. Heteroplasmy frequencies ranged broadly in the *gas-1* MA lines, from as low as 2.3% to complete fixation (homoplasmy). An initially low-frequency (<5%) heteroplasmy discovered in the *gas-1* progenitor was observed to fix in one *gas-1* MA line, achieve higher frequency (37.4%) in another, and be lost in the other three lines. A similar low-frequency heteroplasmy was detected in the N2 progenitor, but was lost in all five N2 MA lines. We identified three insertion-deletion (indel) heteroplasmies in *gas-1* MA lines and six indel variants in the N2 MA lines, most occurring at homopolymeric nucleotide runs. The observed bias toward accumulation of single nucleotide polymorphisms in *gas-1* MA lines is consistent with the idea that impaired mitochondrial activity renders mtDNA more vulnerable to this type of mutation. The consistent increases in mtDNA copy number implies that extreme genetic drift provides a permissive environment for elevated organelle genome copy number in *C. elegans* reference and *gas-1* strains. This study broadens our understanding of the heteroplasmic mitochondrial mutation process in a multicellular model organism.

## Introduction

Nearly all lifeforms observable with the naked eye are composed of eukaryotic cells, most all of which harbor multiple mitochondria (Lane, 2011). These organelles are fundamental to eukaryotic life. In humans, approximately 10% of an individual's body mass is comprised of mitochondria (Nisoli and Carruba, 2006). Mitochondria supply the majority of cellular energy necessary for cell functioning and are central to growth, development, immune response, and reproductive pathways (Dromparis and Michelakis, 2013). Mitochondria respond to cellular stressors (e.g., changes in redox state and substrate availability) and maintain metabolic homeostasis in addition to many other processes necessary for homeostasis (Dromparis and Michelakis, 2013).

The mitochondrial genome is a circular, double-stranded DNA molecule that is nearly always uniparentally inherited—typically from mothers to offspring (Chinnery et al., 2000; Wallace and Chalkia, 2013). Mitochondrial genomes lack histones and many DNA repair systems, potentially contributing to the higher mutation rates observed in mitochondrial DNA (mtDNA) vs. nuclear DNA in metazoans (Denver et al., 2000). Although some mtDNA mutations may be beneficial (Wallace and Youle, 2013), most are known to be deleterious and contribute to neurodegeneration and many debilitating mitochondrial diseases (Kennedy et al., 2012; Wallace and Chalkia, 2013). Previously thought to be rare, mtDNA disorders are now known to affect ~1 in 4300 people (Stewart and Chinnery, 2015). In addition to mutation-generated disease, dysregulation of mtDNA copy number is associated with Alzheimer's Disease and many other pathologies (Lynch et al., 2011; Rice et al., 2014; Pyle et al., 2016). Each mitochondrion contains multiple genome copies (Wallace, 2005), the regulation of which is essential for maintaining proper mitochondrial activity (Lynch et al., 2011). Changes in mtDNA copy number and associated organelle dysfunction may induce mitochondrial retrograde signaling (mitochondria-to-nucleus) as a cellular adaptive response (Guha and Avadhani, 2013). As fluctuations in mtDNA copy number may signify alterations in cellular activity, recent studies in humans and model organisms support the use of mtDNA copy number as a biomarker for aging-related diseases and metabolic syndromes (Lynch et al., 2011; Podlesniy et al., 2013).

Because each cell contains multiple mitochondria, each with multiple genome copies, new mtDNA mutations originate in low-frequency heteroplasmic states; i.e., one copy per cell and organism. We continue to lack a basic understanding of how mtDNA heteroplasmies arise, and how their subsequent frequencies and fates are affected by evolutionary forces operating at different levels (intracellular, between cells, between individuals). Along with nuclear mutations encoding for mitochondrial respiratory chain subunits, single nucleotide mutations in mitochondrial DNA are reported to be a common cause of the childhood disease Leigh syndrome (Chol, 2003; Hadzsiev et al., 2010). Though undetected in parental generations, mitochondrial heteroplasmies may increase in the offspring generation to levels exceeding 80% in both brain and muscle tissues (Hadzsiev et al., 2010). Furthermore, different mitochondrial pathologies are expressed only beyond a critical threshold level (typically 60–80%; Stewart and Chinnery, 2015). The forces controlling the likelihood of transmission of different mtDNA mutation types and their subsequent evolutionary dynamics remain understudied.

Early research investigating mitochondrial variation in natural populations often assumed that all mtDNA molecules within an individual were identical (homoplasmy). Observations of variation in mtDNA, however, reveals that heteroplasmy is a transient though necessary phase during mtDNA evolution (Rand, 2001). Different mtDNA molecules in the heteroplasmic pool may be subject to multi-level selection including selection between mtDNAs in replication, selection at the level of the cell, and selection on the organism (Rand, 2001). This cross-level selection may contest Muller's ratchet by providing a mechanism to purge deleterious mutations (Bergstrom and Pritchard, 1998). In *Drosophilia* smaller mtDNA molecules resulting from VNTR length variation in the origin of replication region have been observed to increase in frequency across generations (Solignac et al., 1987). Similarly, heteroplasmic mtDNA molecules containing large deletions in the *nad5* gene are observed to experience a transmission advantage over larger intact mtDNA molecules in *Caenorhabditis briggsae* (Clark et al., 2012; Phillips et al., 2015). By contrast, the opposite effect has been observed in in the soma of *D. melanogaster* where larger mtDNAs outcompeted smaller ones across the lifespans of female flies (Kann et al., 1998). In mice models past research has determined that although random genetic drift is pervasive in some tissues, in other tissues there is evidence for tissue-specific and age-related directional selection for different heteroplasmic mtDNA genotypes (Jenuth et al., 1997). Given these inconsistent results in a variety of animal study systems, more research is necessary to understand the evolutionary dynamics of mtDNA heteroplasmy.

*C. elegans* provides an outstanding model for examining mitochondrial biology. The nuclear and mitochondrial genomes are well-characterized; a variety of genetic mutants and other community resources enable diverse investigations into mitochondrial genetic and physiological questions. Additionally, Illumina-MiSeq can be applied to evolved lines to detect heteroplasmy and assess relative levels of heteroplasmic to wild-type mtDNA. Sanger sequencing can also be applied to individual worms to detect the presence of heteroplasmies. Past research employing the *C. elegans* system demonstrated increases in mtDNA copy number over development, suggesting that mtDNA might have variable effects across nematode lifespan (Tsang and Lemire, 2002; Bratic et al., 2009, 2010). Further, *C. elegans* mtDNA copy number increases up to three-fold in response to diet and environmental stimuli (Reinke et al., 2010). Thus, mtDNA copy number is known to be plastic within a nematode generation. However, little is known about the trans-generational plasticity of, and effects of fundamental evolutionary forces (e.g., genetic drift, natural selection), on mtDNA copy number in *C. elegans* or any other animal system. Past work employing the *C. elegans* system and mutation-accumulation (MA) approaches has improved our understanding of the rate and spectrum of mtDNA changes across generations (Denver et al., 2000, 2009; Tsang and Lemire, 2002). Importantly, work in this system revealed that the baseline rate of mtDNA mutation was two orders of magnitude higher than previously thought and identified homopolymer nucleotide stretches and repeat sequences as insertion-deletion (indel) mutational hotspots, similar to mtDNA variation associated with human pathologies (Denver et al., 2000). Furthermore, mutation patterns analyzed in *C. elegans* MA lines demonstrated a dominant role for purifying natural selection in shaping mtDNA protein-coding genes in natural populations (Denver et al., 2012).

We employed a MA approach using a well-characterized *C. elegans* mutant, *gas-1*, to study the impact of mitochondrial dysfunction on mtDNA variation. The *C. elegans gas-1* gene is nuclear-encoded and orthologous to human *NDUFS2* (83.4% protein similarity). *gas-1* encodes a 49 kDa subunit of Complex I of the mitochondrial electron transport chain (ETC). The *gas-1* fc21 allele, used here, is a single-base replacement mutation that renders the NAD-1 protein subunit dysfunctional, resulting in significantly increased endogenous reactive oxygen species (ROS) production, along with decreased ATP (Hartman et al., 2001; Kayser et al., 2004; Pujol et al., 2013). The *gas-1* mutation reduces ETC Complex I efficiency by 75%, a mitochondrial phenotype commonly observed in many neurodegenerative pathologies such Alzheimer's and Parkinson's Disease (Vasta et al., 2011; Schulz et al., 2012). Moreover, Complex I deficiencies are the most common cause of mitochondrial disease in both adults and children. *NDUFS2* mutations are reported in fatal infantile lactic acidosis (FILA), Leigh Syndrome, and hypertrophic cardiomyopathy (Falk et al., 2009; Tuppen et al., 2010; Van den Ecker et al., 2012; Pujol et al., 2013). While other *C. elegans* mitochondrial mutants such as *isp-1* and *cyc-1* exhibit extended lifespan in comparison to wild-type, published values for the *gas-1* report a significant decrease in lifespan (Hartman et al., 2001; Kayser et al., 2004; Van Raamsdonk and Hekimi, 2011; Pujol et al., 2013). *gas-1* nematodes have reduced fecundity, delayed growth rates, and exhibit hypersensitivity to oxidative stress as shown by both hypoxia and paraquat exposure (Hartman et al., 2001; Kayser et al., 2001). Employing the *gas-1* strain therefore provides a disease-relevant context for studying mtDNA variation. We applied Illumina MiSeq technology to analyze five different *gas-1* MA lines that were bottlenecked for an average of 43 generations, alongside five N2 MA lines from a previous study, bottlenecked for 250 generations (Baer et al., 2005). Bioinformatic analysis identified mitochondrial genomic changes including mtDNA copy number shifts, and heteroplasmic single-base substitutions and indels.

## Materials and methods

### Strains and backcrossing of *gas-1* mutant

This study utilized the *gas-1* (fc21) mutant containing a C → T point mutation that replaces a highly conserved arginine with lysine in the GAS1 protein, a central component of mitochondrial ETC Complex I. Because *gas-1* (fc21) originated from an ethyl methanesulfonate (EMS) mutagenesis screen, the strain was likely to contain many other mutations (Kayser et al., 1999). We therefore backcrossed the *gas-1* strain, CW152, obtained from the *Caenorhabditis* Genetics Center (University of Minnesota) to our wild-type N2 strain for 10 generations to create an isogenic mutant strain. Briefly, a N2 male was first mated to a *gas-*1 hermaphrodite, producing *gas-1* heterozygous progeny at the F1 generation. Several single heterozygous hermaphrodite offspring were isolated and mated with a N2 male, producing the F2 generation. The F2 progeny were screened for the presence of the *gas-1* mutation using PCR and direct Sanger sequencing of the amplicon (forward primer: ATCTCCTCAATACGGCACAAG; reverse primer: ATCGTCTCGATTACGTCTCCA), and only hermaphrodites retaining the *gas-1* mutation were maintained. This sequencing confirmation was continued for backcross generations F3-F10 and only *gas-1* heterozygous hermaphrodites were used at each crossing. After 10 sequential backcrosses, the resulting *gas-1* heterozygous lineages were allowed to self and the offspring were screened to find nematodes homozygous for the fc21 allele. These *gas-1* homozygous lineages were used to initiate MA lines. All nematodes were cultured under standard laboratory conditions at 20°C on standard NGM agar plates seeded with OP50 *Escherichia coli*.

### Nematode strains and culture conditions

We studied two sets of MA lines generated from either a *gas-1* mutant or a Bristol N2 wild-type (G0) progenitor. A set of 48 *gas-1* MA lines was derived from the offspring of a single backcrossed *gas-1* hermaphrodite. Lines were maintained by transferring single fourth-larval stage (L4) offspring to fresh plates at 4-day intervals for an average of 43 generations (35–47 MA generations; G43 MA) (Supplementary Figure 1), after which time they were stored cryogenically at −80°C (Stiernagle, 2006). The MA process minimizes the power of natural selection and maximizes genetic drift, allowing all but the most highly deleterious mutations to accumulate in an effectively neutral fashion (Halligan and Keightley, 2009). At each transfer, the prior generation was maintained at 10°C. In the case of a sterilizing or lethal mutation, a replacement L4 nematode was selected at random from these “backup” plates. This resulted in fewer generations propagated for some MA lines (Supplementary Table 1). We also utilized five MA lines generated from N2 as part of a previous experiment (Baer et al., 2005), and for which mutational declines in fitness, whole-nuclear genome sequence, ROS, and oxidative DNA damage levels are available (Denver et al., 2009; Clark et al., 2012; Joyner-Matos et al., 2013). These MA lines were initiated from the offspring of a single, highly inbred N2 hermaphrodite and evolved in the manner described above for an maximum of 250 generations (G250 MA) (Baer et al., 2005).

### L1 stage DNA preparation for Illumina-MiSeq MA lines

Twelve nematode strains—five randomly selected *gas-1* MA lines, five previously-studied N2 MA lines (Baer et al., 2005), and their respective ancestral strains, N2 and the backcrossed *gas-1*—were developmentally synchronized by bleaching according to standard protocols (Wood, 1988). First larval (L1) stage nematodes were harvested and DNA was purified using Qiagen DNeasy Blood & Tissue kit (Valencia, CA) with one modification to the manufacturer's protocol. Prior to adding AL buffer, worms in a solution of M9 buffer, ATL, and Proteinase K were subjected to five cycles of freezing and thawing to break worm cuticles and allow efficient DNA extraction. DNA was prepared for sequencing using standard Illumina TruSeq protocols for genomic DNA. Samples were individually barcoded and pooled for two runs (one per a background), both sequenced using the Illumina MiSeq Genome Analyzer operated by the Oregon State University Center for Genome Research and Biocomputing. All sequences were deposited to NCBI under SRA: accession number SRP069774.

### Illumina-MiSeq read mappings and analyses

Following each IlluminaMiSeq run, reads were aligned to the *C. elegans* genome (version WS242) using CLC Genomics Workbench (CLC Bio-Qiagen, Aarhus, Denmark). All reads were paired-end (2 × 150 bp) and mapped using the following parameters: No masking, mismatch cost = 2, insertion cost = 3, deletion cost = 3, length fraction = 0.98, read fraction = 0.98, global alignment = no, non-specific match handling = map randomly.

### Bioinformatic analyses of mtDNA copy number

mtDNA copy number was normalized by nuclear DNA (nDNA) content. Specifically, relative mtDNA for each line was calculated as the line-specific average mtDNA coverage divided by the line-specific average coverage of three single-copy nuclear genes: *ama-1, ego-1*, and *efl-2*. The AT-rich region of the mitochondria genome was not considered in these calculations due to inconsistencies during sequencing created by its repetitive nature (Table 1).

**Table 1:** **Illumina-MiSeq sequencing run statistics and normalization of mean mtDNA copy number**.

**Line**	**Total reads**	**% Mapping**	**Avg Nuc Cov**	**Avg mtDNA Cov**	**Norm mtDNA**	**Avg single-copy**	**Avg *ama-1* Cov**	**Avg e*go-1* Cov**	**Avg *efl-3* Cov**
**N2**									
Progenitor	12,474,086	87.7	27.3(54.7)	330.7(128.0)	9.4	37.0	36.3(13.7)	39.0(11.4)	35.7(14.2)
MA523	8,162,820	92.7	11.3(15.1)	241.6(62.8)	19.8	12.2	10.4(3.3)	13.1(3.5)	13.1(3.1)
MA526	6,698,142	89.5	8.9(13.1)	636.2(85.7)	71.1	9.0	7.4(2.7)	9.3(3.4)	10.2(4.4)
MA529	6,799,824	93.0	9.4(12.2)	414.2(52.8)	42.4	9.8	8.9(3.2)	10.1(3.2)	10.3(3.0)
MA553	7,846,166	92.0	10.7(15.9)	569.1(74.1)	51.4	11.1	9.1(3.6)	12.1(4.6)	12.0(4.5)
MA574	6,271,490	93.2	8.7(12.4)	449.5(67.9)	50.1	9.0	7.1(3.3)	9.2(4.3)	10.7(4.1)
***gas-1***									
Progenitor	10,725,726	88.6	23.7(36.9)	221.3(86.2)	7.2	30.8	26.2(9.6)	35.0(9.2)	31.2(11.2)
MA412	7,558,954	83.4	15.6(14.5)	324.5(79.9)	18.0	18.1	13.8(5.5)	20.3(5.8)	20.1(7.3)
MA419	6,843,258	82.5	14.0(13.7)	308.9(99.9)	28.3	10.9	10.6(4.3)	14.9(4.2)	7.3(5.9)
MA429	7,351,108	86.8	15.9(12.3)	498.8(74.2)	29.1	17.2	11.7(5.0)	20.0(6.8)	19.8(6.2)
MA431	6,896,562	80.6	13.7(13.7)	286.6(98.4)	19.4	14.8	10.9(4.2)	17.4(4.5)	15.9(6.8)
MA438	6,998,936	83.4	14.4(17.4)	259.6(100.1)	17.1	15.2	11.1(4.8)	17.3(5.6)	17.1(6.4)

*All reads 150 bp, paired end. All nematodes at L1 stage. Assembled using WS242 C. elegans reference genome in CLC with following parameters: no masking, mismatch cost = 2, insertion cost = 3, deletion cost = 3, length fraction = 0.98, read fraction = 0.98, global alignment = no, non-specific match handling = map randomly. mtDNA copy number normalized by mean coverage of corresponding single-copy nuclear genes (Norm mtDNA). mtDNA mean coverage calculated excluding AT-region (Avg mtDNA Cov). Overall nuclear coverage (Avg Nuc Cov) calculated for entire nuclear genome per a line. Average single-copy nuclear coverage (Avg Single-Copy) calculated from average of three single copy genes: ama-1(Avg ama-1 Cov), ego-1(Avg ego-1 Cov), efl-3 (Avg efl-3 Cov). Standard deviation of the mean displayed in parentheses adjacent to mean values*.

We tested normalized mtDNA copy number data for normality and consistent variance. A Levene's test determined that the data had inconsistent variance (*P* = 3.3 × 10^−16^) among lines, and a box and whiskers plot displayed the non-normality of the data (Supplementary Figure 1). We applied the non-parametric Kruskal Wallis H Test with strain as an explanatory variable to evaluate if there was a statistically significant increase in mtDNA copy number following bottlenecking. Using normalized mitochondrial DNA coverage for 12 mitochondrial genes and two ribosomal sequences in each of the progenitors and respective five MA lines, the analysis determined strain was a significant factor (*P* = 2.2 × 10^−16^, Kruskal Wallis H Test; Supplementary Table 2). *Post-hoc* analysis applied a Kruskal Multiple Comparison test in order to evaluate the significance of mtDNA copy number among pairwise comparisons of all lines (Supplementary Table 3).

### Bioinformatic identification of mtDNA variants

Potential line-specific mtDNA variant sites were identified as mitochondrial genome positions differing from the *C. elegans* reference genome (WS242) and not fixed within our wild-type N2 lab strain. Site-specific variant frequency was calculated by dividing the number of variant calls by the total site coverage. To eliminate false positives resulting from sequencing artifacts, at least six variant calls were required for a variant to be considered a potential mtDNA heteroplasmy. Furthermore, sites were required to be within one standard deviation of the line-specific per-site mean mitochondrial coverage and to have a variant frequency greater than two standard deviations above the line-specific mean variant frequency to be considered a mtDNA heteroplasmy (Tables 2, 3).

**Table 2:** **Mitochondrial heteroplasmic single-nucleotide polymorphisms**.

**Line**	**Pos**	**Gene**	**Ref Base**	**Ref Counts**	**Var Base**	**Var Counts**	**Var Freq**	**Ref Codon**	**Ref AA**	**Var Codon**	**Var AA**	**Type**
**N2**												
Progenitor	8274	*COX-I*	G	495	A	21	0.041	GGG	Gly	GAG	Glu	Non-Syn
MA523	8731	*COX-I*	C	292	A	18	0.058	GCT	Ala	GAT	Asp	Non-Syn
***gas-1***												
Progenitor	8439	*COX-I*	G	344	T	18	0.050	GTG	Val	TTG	Leu	Non-Syn
MA412	8439	*COX-I*	G	259	T	155	0.374	GTG	Val	TTG	Leu	Non-Syn
MA431	211	*NAD-6*	A	238	T	9	0.036	CTA	Leu	CTT	Leu	Syn
	450	*NAD-6*	A	140	T	6	0.041	CTA	Leu	CTT	Leu	Syn
	5422	*Cyt-b*	A	261	T	7	0.026	ACC	Thr	ACT	Thr	Syn
	7176	*NAD-4*	T	312	C	36	0.103	TTA	Leu	TCA	Ser	Non-Syn
	11965	*NAD-5*	T	203	A	6	0.029	CTA	Leu	CAA	Gln	Non-Syn
MA438	211	*NAD-6*	A	252	T	6	0.023	CTA	Leu	CTT	Leu	Syn
	8439	*COX-I*	G	0	T	447	1.000	GTG	Val	TTG	Leu	Non-Syn

Ref Base, Reference Allele; Var Base, Variant Allele; Ref Codon, Reference Codon; Ref AA, Reference Amino Acid; Var Codon, Variant Codon; Var AA, Variant Amino Acid. Site specific variant frequency (Var Freq) calculated by dividing number of variant calls (Var Counts) by the total coverage at the position (Var Counts + Ref Counts). SNPs required to be within one standard deviation of the line-specific per-site mean mitochondrial coverage, have a variant frequency greater than two standard deviations above the line-specific mean variant frequency and at least six variant calls per SNP.

**Table 3:** **Mitochondrial heteroplasmic indel variants**.

**Line**	**Pos**	**Gene**	**Ref Allele**	**Ref Counts**	**Var Allele**	**Var Counts**	**Var Freq**	**Homopoly Len**	**Type**
**N2**									
MA523	3235	*ATP6*	A	211	–	11	0.0495	11	Deletion
	11721	*NAD-5*	–	193	T	13	0.063	8	Insertion
MA526	3235	*ATP6*	A	113	–	441	0.796	11	Deletion
	8653	*COX-I*	–	611	A	11	0.018	2	Insertion
MA529	3235	*ATP6*	A	372	–	15	0.0388	11	Deletion
MA553	3235	*ATP6*	A	493	–	21	0.041	11	Deletion
MA574	3235	*ATP6*	A	255	–	136	0.348	11	Deletion
	11721	*NAD-5*	–	236	T	152	0.392	8	Insertion
***gas-1***									
Progenitor	9954	*tRNA-C*	T	235	–	11	0.0447	2	Deletion
MA419	3235	*ATP6*	A	370	–	11	0.029	11	Deletion
MA429	3235	*ATP6*	A	502	–	25	0.047	11	Deletion
MA431	3235	*ATP6*	A	375	–	17	0.043	11	Deletion
MA438	3235	*ATP6*	A	321	–	11	0.033	11	Deletion

Site specific variant frequency calculated by dividing number of variant calls by the total coverage at the position. Indels required to be within one standard deviation of the line-specific per-site mean mitochondrial coverage, have a variant frequency greater than two standard deviations above the line-specific mean variant frequency and at least six variant calls per indel. Dashes in the reference allele column (Ref Allele) indicate an insertion and that the base missing from reference genome. Dashes in variant allele column (Var allele) indicate a deletion; base missing from experimental line.

### Sanger sequencing assay of bulk and individual nematodes

Sanger sequencing was applied to individual and bulk L1 nematodes to screen for presence of a specific heteroplasmic site, initially detected by Illumina sequencing, occurring at low-frequency in the *gas-*1 progenitor and in three of its descendant *gas-1* MA lines (Supplementary Tables 3–9). Analysis of L1 individuals should provide the most accurate reflection of inherited levels of mtDNA heteroplasmy since these nematodes have not yet undergone the mtDNA copy number expansions occurring at L4 and young adult stages (Tsang and Lemire, 2002). For all bulk extractions, DNA was harvested from L1 worms and purified as described above. DNA from individual L1 nematodes was harvested from single worms placed in standard worm lysis buffer solution prior to the five freeze-thaw cycles (Williams et al., 1992). Following DNA extraction, mitochondrial DNA was amplified using two primers flanking the heteroplasmic site of interest (forward primer: TCGGTGGTTTTGGTAACTGA; reverse primer: CAACACCTGTCAACCCACCT). PCR products were purified through solid phase reversible immobilization (SPRI) techniques(Elkin et al., 2001). The amplicon was Sanger sequenced using an internal primer: AGCAGCCGAAAAATAAGCAC.

**Figure 1:**
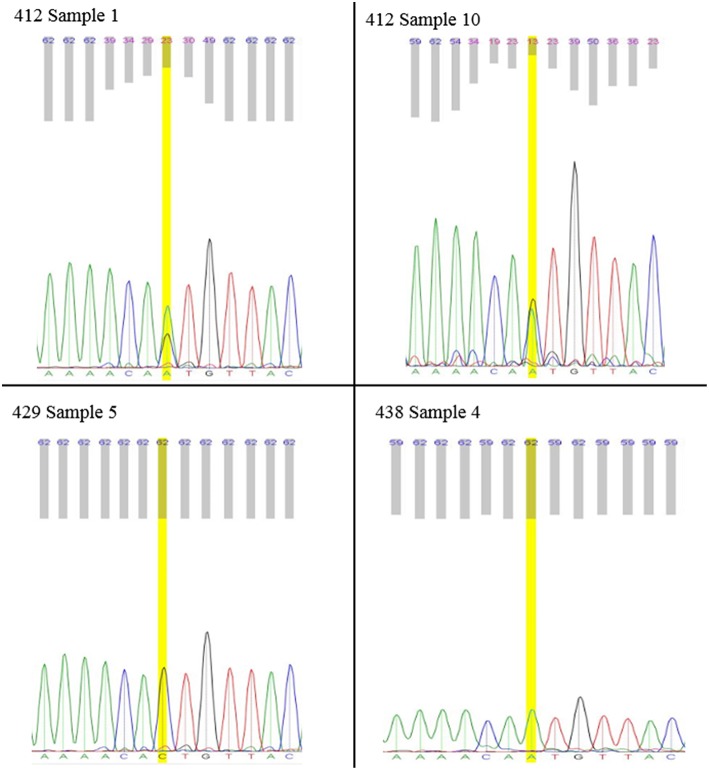
**Individual L1's Sanger Electropherogram**. Sanger results for mitochondrial position 8439 heteroplasmy of individual *gas-1* worms. Electropherograms for MA412 samples display presence of both “A” and “C” alleles. MA429 is, fixed for the wild-type “C” allele. MA438 is fixed for the alternate “A” allele. Lower phred scores, displayed above electropherograms, indicate uncertainty in base calling associated with heteroplasmic sites.

A total of 18 *gas-1* progenitor worms, 17 *gas-1* MA 412 worms, 18 *gas-1* MA 429 worms, and 17 *gas-1* MA 438 worms were individually assayed (Supplementary Tables 3–6). Bulk extractions were harvested from both the *gas-1* and N2 progenitor in addition to 14 *gas-1* MA lines (Supplementary Tables 8, 9). Phred scores were used to indicate the uncertainty of Sanger sequencing in identifying a specific base in a heteroplasmic state. No candidate heteroplasmic sites were excluded on the basis of Phred score; Phred scores were used to validate heteroplasmies that evaluated by Sanger sequencing. We used low-quality Phred scores as an indicator of heteroplasmy, reflecting the presence of more than one nucleotide signal. To ensure that low Phred scores were a product of heteroplasmy rather than poor sequence quality, Phred scores for the heteroplasmic site were compared to the average Phred score for the 50 base pairs flanking the heteroplasmy in both directions (Figure 1).

## Results

To investigate mtDNA mutation and heteroplasmy, we analyzed mtDNA genomes of the N2 and backcrossed *gas-1 C. elegans* progenitors, five randomly selected *gas-1* MA lines, and five previously-studied N2 MA lines using Illumina MiSeq technology (Denver et al., 2009, 2012). In all lines, over 80% of reads mapped to the *C. elegans* WS242 reference genome with a mean nDNA coverage of 14.5X and a mean mtDNA coverage of 378.4X per line (Table 1). Analysis of Illumina nDNA sequence data showed that all *gas-1* MA lines remained homozygous for the *gas-1* mutant allele. Analyses of nuclear genome mutation from these lines will be reported in a separate paper.

### Changes in mtDNA copy number

Relative mtDNA copy number (mtDNA coverage normalized by coverage at single-copy nuclear regions—see Materials and Methods for details) was not statistically different between the N2 and *gas-1* progenitor strains. (9.4 and 7.2, respectively; Supplementary Table 3). Three *gas-1* MA lines (MA412, MA431, and MA438) as well as one N2 MA line (MA523) were not significantly different than the *gas-1* and N2 progenitors (Supplementary Table 3). The six remaining MA lines MA419, MA429, MA526, MA529, MA553, and MA74 showed significant increases in relative mtDNA copy number compared to their respective progenitor strains (Supplementary Table 3). In the *gas-1* MA lines, mean normalized mtDNA copy number was 22.4X, or on average three-fold higher than in the *gas-1* progenitor (range = 2.2–3.4X higher). Similarly, mean relative mtDNA copy number for the N2 MA lines was 47.7X, five-fold higher than in the N2 progenitor (range = 1.8–6.0X higher). Coverage patterns across sites were consistent among lines, indicating that mtDNA copy number had increased at all sites (i.e., increases in complete mitogenome copy number) rather than at isolated sections of the mitochondrial genome.

### mtDNA variants

We identified and characterized all mitochondrial variants (both heteroplasmic and fixed) in all progenitor and MA lines. Candidate heteroplasmic single-nucleotide polymorphisms (SNPs) were filtered to eliminate false-positives (see Materials and Methods). Compared to the *C. elegans* reference genome, the N2 progenitor harbored a low-frequency heteroplasmic base substitution in the *COX-1* gene (Table 2); this SNP was lost in all of the five N2 MA lines analyzed. A *tRNA-C* deletion was lost in all five *gas-1* MA lines analyzed, while a *gas-1* progenitor *COX-I* heteroplasmy was transmitted to some MA lines (discussed in more detail below). This *COX-1* SNP detected in the N2 progenitor was not detected in the *gas-1* progenitor or its MA lines; however, the *gas-1* progenitor did contain a low-frequency heteroplasmy at a different *COX-I* site in addition to a single-nucleotide deletion in the *tRNA-C* gene (Table 3).

**Figure 2:**
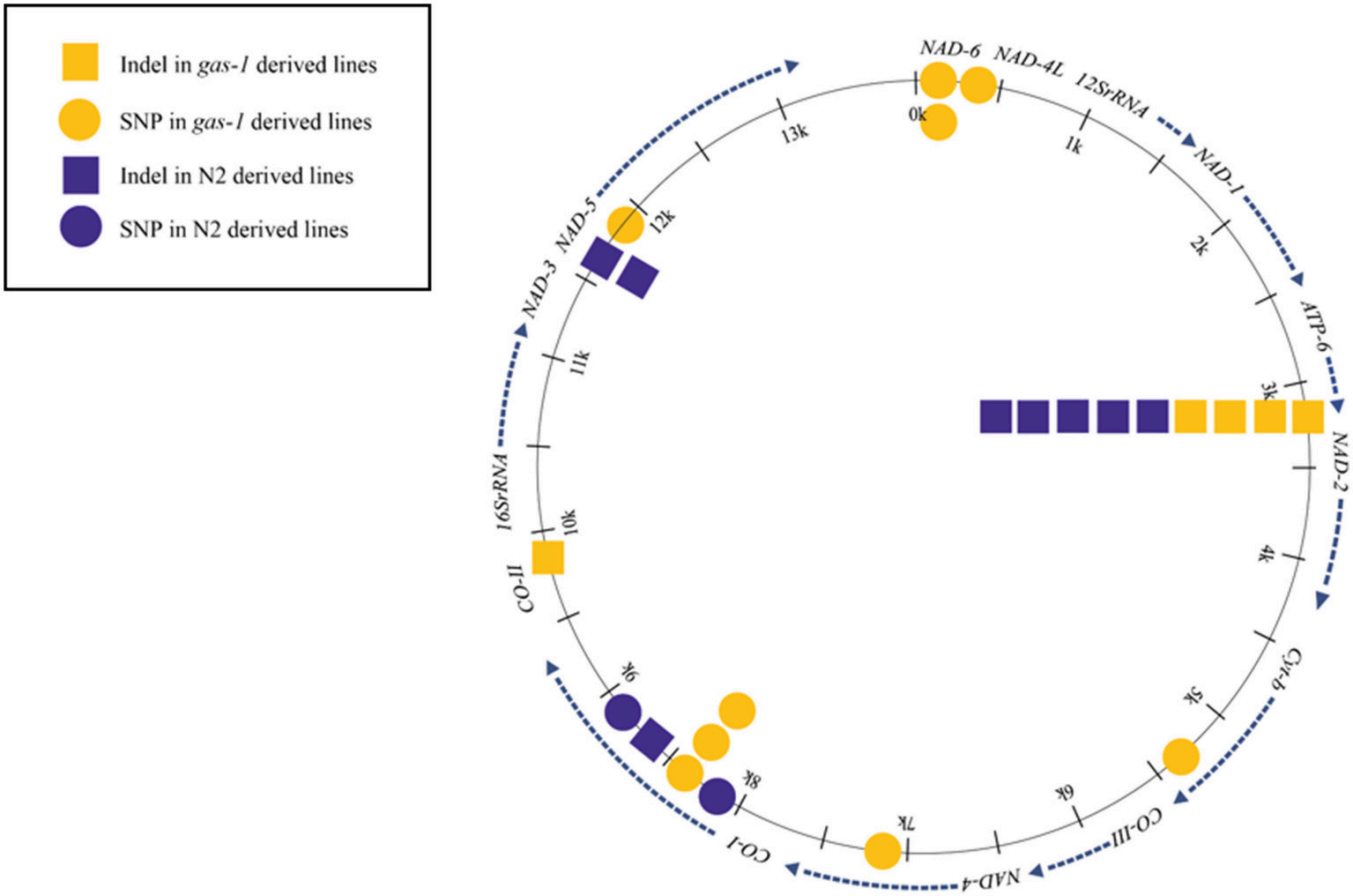
**Schematic of Mitochondrial Indel and Single-Base Substitution Heteroplasmies**. All indel and single-base substitution events for all wild-type N2 and *gas-1* lines.

Twelve unique heteroplasmic variants were identified in the *gas-1* MA line set: seven SNPs and five indels (Tables 2, 3). In the case of the heteroplasmy at position 211, where MA431 and MA438 experienced an identical heteroplasmy event, the heteroplasmy was counted twice. Likewise, as four *gas-1* MA lines experienced an indel event at position 3235 in the *ATP6* gene, this event was counted four times. In contrast, when the heteroplasmy was present in the progenitor, the event was only counted once. The *COX-I* (position 8439) variant present in the *gas-1* progenitor was lost (or present at undetectable levels) in three *gas-1* MA lines and inherited by the remaining two. Of the two lines that inherited this SNP, one (MA412) harbored it at moderate levels (37%), and the other (MA438) harbored it at 100% (homoplasmic) frequency (Table 2). Of the new (undetected in progenitors) variants in the *gas-1* MA lines, two were located in the *NAD-6* gene. One of these was shared by two *gas-1* MA lines (MA431 and MA438) and present at similarly low levels—3.6 and 2.3%, respectively (Tables 2, 4). Five of the *gas-1* MA-specific SNP heteroplasmies were present in a single *gas-1* MA line, MA431; these include the two occurring in the *NAD-6* gene and one in each of the *cyt-b, NAD-4*, and *NAD-5* genes. The *NAD-4* heteroplasmy was present at the highest frequency (10%). Only one mtDNA indel (a single bp deletion), occurring within a homopolymeric run within the *ATP6* gene, was detected in the *gas-1* MA line set. This variant was shared among four of the five *gas-1* MA lines at low frequency (<5%) (Tables 3, 4; Figure 2).

**Table 4:** **SNPs and indels frequencies for all mitochondrial sites containing variants**.

**Site**	**N2 Prog**	**MA523**	**MA526**	**MA529**	**MA553**	**MA574**	***gas-1* Prog**	**MA412**	**MA419**	**MA429**	**MA431**	**MA438**
**SNPs**												
211	0.008	0.003	0.004	0.006	0.002	0.002	0.000	0.011	0.020	0.018	**0.036**	**0.023**
450	0.000	0.000	0.000	0.008	0.000	0.000	0.007	0.000	0.006	0.002	**0.041**	0.007
5422	0.000	0.000	0.008	0.004	0.000	0.002	0.006	0.008	0.009	0.004	**0.026**	0.013
8274	**0.041**	0.000	0.001	0.002	0.000	0.000	0.000	0.000	0.008	0.000	0.002	0.000
8439	0.002	0.000	0.000	0.000	0.000	0.000	**0.050**	**0.374**	0.000	0.000	0.000	**1.000**
8731	0.001	**0.060**	0.002	0.000	0.000	0.002	0.000	0.000	0.000	0.002	0.000	0.000
**INDELS**												
3235	0.021	**0.050**	**0.796**	**0.038**	**0.041**	**0.348**	0.013	0.021	**0.029**	0.047	**0.043**	**0.033**
8653	0.001	0.003	**0.018**	0.000	0.000	0.000	0.003	0.002	0.000	0.000	0.002	0.000
9954	0.002	0.000	0.000	0.000	0.000	0.000	**0.045**	0.000	0.000	0.000	0.000	0.002
11721	0.000	**0.063**	0.090	0.002	0.000	**0.392**	0.000	0.000	0.003	0.002	0.004	0.000

Each site which experienced a variant in any of the 12 lines is listed by reference position in WS242 (base pair). Frequencies which appear in Tables 2, 3 are in bold. A value of “0” indicates no variant allele occurred for at this site for the line.

Nine mtDNA heteroplasmies were detected in the five N2 MA lines—one SNP in the *COX-I* gene and eight indel mutations (Tables 2, 3). The new SNP heteroplasmy in *COX-1* was found in only one N2 MA line, MA523. Conversely, every N2 MA line contained at least one indel, the majority comprising single-bp deletions compared to the reference genome (Table 3). All five N2 MA lines analyzed contained the same *ATP6* variant detected in four of the *gas-1* MA lines (Figure 2) at frequencies ranging from 4–80% (Tables 3, 4). In addition to the *ATP6* variant, N2 MA526 also contained a single-bp insertion in the *COX-I* gene while MA523 and MA574 shared the same heteroplasmic insertion variant located at 11,721 bp in the *NAD-5* gene. Most indel variants in the N2 MA lines were present at very low frequencies (<7%) (Tables 3, 4).

### Inheritance patterns of *gas-1* progenitor SNP heteroplasmy

Bioinformatic analysis of Illumina sequence for the *gas-1* MA lines set revealed variable inheritance outcomes for the heteroplasmic SNP in *COX-1*, initially present in the *gas-1* progenitor at about ~5%: (1) Loss or dramatically reduced frequency in three of the five MA lines, (2) increased frequency (up to 37%) in MA412, and (3) complete fixation at 100% in MA438 (Table 2). To investigate the inheritance patterns of this heteroplasmic SNP on a finer scale, Sanger sequencing was applied to survey for its presence in many individual L1 nematodes of the *gas-1* progenitor (Supplementary Table 4) and from three of the five sequenced *gas-1* MA lines (Supplementary Tables 3–5). Due to the nature of the Sanger sequence data, we were unable to quantify heteroplasmic frequencies, but instead characterized our results based on three electropherogram categories: SNP absent, SNP fixed, and SNP still segregating with wild-type base.

The presence of this focal heteroplasmy varied among individual *gas-1* progenitor nematodes; the heteroplasmy was absent in some individuals and still segregating or fixed in others (Supplementary Table 4). Analysis of individual nematodes from *gas-1* MA412, MA429, and MA438 lines revealed substantial variation among MA lines in the frequency of the 8439 heteroplasmy, but far less inter-individual (or intra-line) variation compared to the *gas-1* progenitor. Namely, in agreement with Illumina sequence results (Tables 2, 4), all MA412 worms analyzed harbored the 8439 heteroplasmy in addition to wild-type genomes (Figure 1; Supplementary Table 3), all MA429 worms lacked the 8439 heteroplasmy (Figure 1; Supplementary Table 6), and all MA438 worms were homoplasmic for the variant (Figure 1; Supplementary Table 7).

To further explore the heteroplasmic inheritance patterns of the 8439 *COX-I* heteroplasmy, we applied Sanger sequencing to bulk DNA extractions of nematodes from an additional 11 *gas-1* MA lines alongside the *gas-1* and N2 progenitors. This heteroplasmy was found to be fixed in two of the 11 lines, present as a still-segregating heteroplasmic variant in two others, and lost or undetectable in the remaining seven MA lines (Supplementary Table 9).

## Discussion

Our study reports observed patterns of mitochondrial genome change in wild-type (N2) and mitochondrial ETC mutant (*gas-1*) *C. elegans*, evolving in the lab under extreme drift. Increased mtDNA copy number was consistently observed in all MA lines initiated from both strains. Of the 13 indels identified across MA lines (including instances of parallel indel heteroplasmy observed in more than one line), the majority occurred in N2 MA lines. Of the 11 total SNPs identified (again including instances of parallel changes), the majority occurred in *gas-1* MA lines, which evolved for less than one fifth the number of MA generations experienced by the wildtype N2 lines. The application of high-throughput DNA sequencing allowed us to gain insights into mitochondrial genome copy number changes, and to detect low-frequency heteroplasmic variants not possible with PCR/Sanger sequencing approaches applied in the past (Denver et al., 2000).

### mtDNA copy number

**Figure 3:**
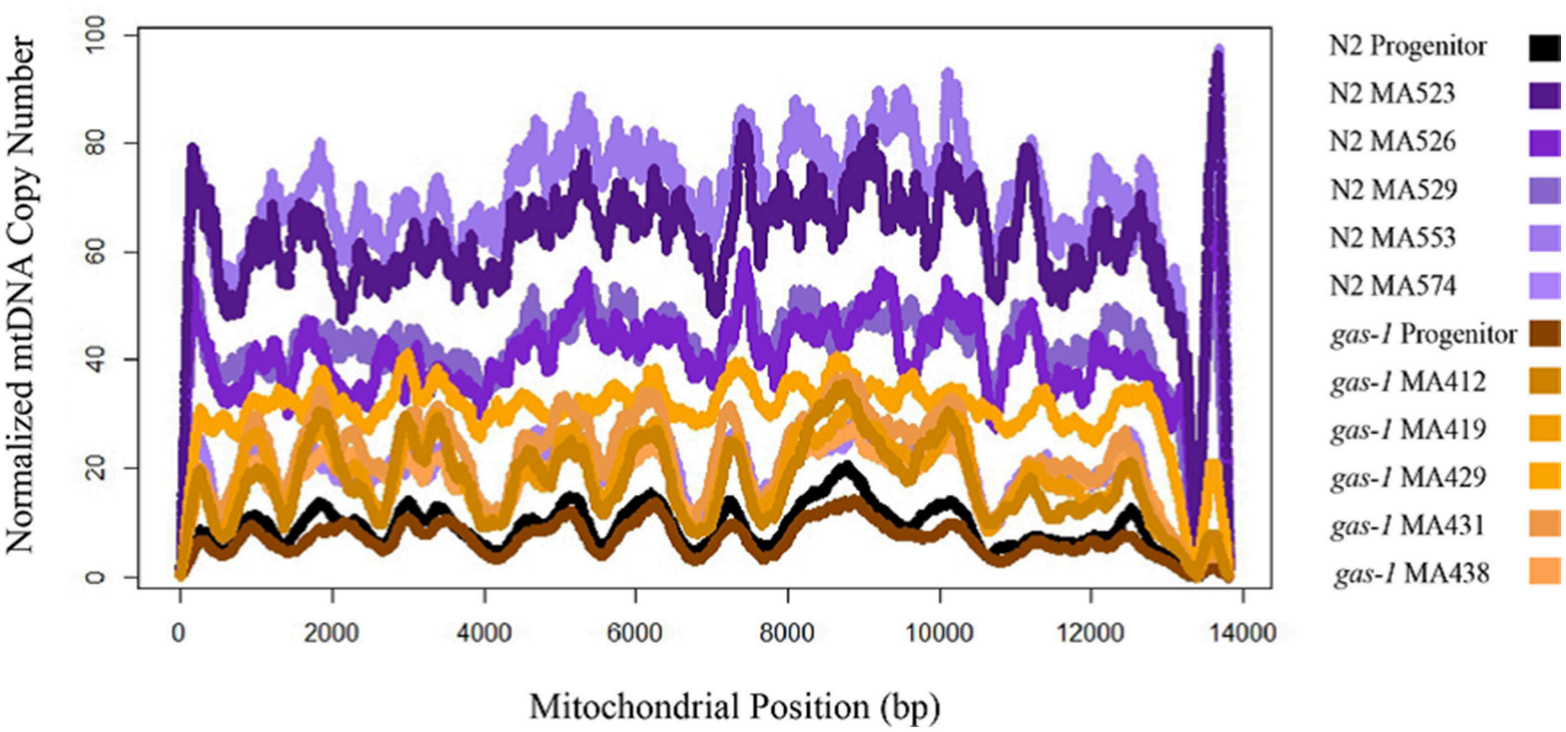
**mtDNA copy number N2 and *gas-1* progenitor vs. MA lines**. All nematodes at L1 stage. N2 MA lines bottlenecked for 250 generations. *gas-1* MA lines bottlenecked for a maximum 50 generations. mtDNA copy number normalized by corresponding line-specific coverage of single-copy nuclear genes: *ama-1, ego-1*, and *efl-3*. N2 MA lines mean mtDNA copy number = 47.43; standard deviation = 19.9. *gas-1* MA mean mtDNA copy number = 12.89, standard deviation 5.63. The AT-rich region was excluded from analysis.

Our observation that N2 and *gas-1* derived MA lines both experienced increased mtDNA content across generations suggests that single-worm bottlenecking may lead to elevated mtDNA copy number in *C. elegans* wildtype and *gas-1* strains (Figure 3). All MA lines were observed to increase in mtDNA copy number relative to the respective progenitor strain.Two of out five *gas-1* MA lines and four out of five N2-derived MA lines had significant changes in mtDNA copy as determined by Kruskal Multiple Comparison's test. N2-derived MA lines experienced bottlenecking for 250 generations and exhibited an average five-fold increase in mtDNA relative to the wild-type progenitor, which translates to a ~2% increase in mtDNA copy number per a generation. Similarly, the *gas-1* derived MA-lines were bottlenecked for an average 43 generations, experienced on average a three-fold elevation in mtDNA copy number which translates to a ~150% increase in mtDNA copy number per a generation. No *gas-1* MA lines actually achieved G50. An important caveat to interpreting our mtDNA copy number results is that interim generational values for mtDNA quantity were not obtained and further experimentation would be needed to address any occurrence of fluctuating mtDNA copy number across generations. Our trans-generational analysis demonstrates that mtDNA copy number can increase across generations in *C. elegans* wildtype and *gas-1* strains, adding to previous studies showing that mtDNA copy number increases during development within a single *C. elegans* generation (Bratic et al., 2009). Furthermore, our results were observed for *C. elegans* reference and *gas-1* strains and may not extrapolate to other *C. elegans* strains.

The evolutionary and molecular causes underlying the variation we observed in mtDNA copy number are not fully understood, although our study suggests three hypotheses: increased oxidative stress, extreme drift, and smaller mtDNA molecules. As past research has suggested that oxidative damage to DNA may result in increased mtDNA copy number, particularly in certain cell types (i.e., leukocytes), one hypothesis is that oxidative damage may be contributing to increases in mitogenome copy number (Liu et al., 2003). The *gas-1* progenitor exhibits elevated endogenous ROS levels and a study conducted on N2 MA lines demonstrated that these lines evolved higher endogenous ROS levels due to bottlenecking (Joyner-Matos et al., 2013). Because both the *gas-1* progenitor and N2 MA lines exhibit elevated ROS levels, future work studying wild-type MA lines at earlier generations (where ROS levels might be lower) will be required to disentangle the influences of drift and oxidative damage on mtDNA copy number increase. Alternatively, there could be a break down in retrograde signaling during mutation accumulation that could initiate a feedback loop generating increased copy number. Due to the extreme drift imposed on *C. elegans* and lack of competition for resources during bottlenecking, the quality-control mechanism eliminating mitochondrial genomes may have been disturbed. The extreme drift on the organismal level may have imposed downstream effects on the level of mitochondria resulting in relaxed selection on mtDNA populations within the organelle and causing massive imbalance of mtDNA content in cells. Extreme drift may eliminate selection on the level of the mitochondrion, creating an imbalance in mitochondrial quality-control and lead to increased mtDNA copy number to compensate for dysfunctional mitochondrial genomes in *C. elegans* N2 wildtype and *gas-1* mutant strains. A third hypothesis is that smaller mtDNA molecules may have evolved. As our analysis excluded the AT-rich region located after the tRNA-alanine sequence, we are unable to detect if the AT-region may have experienced deletions resulting in smaller molecules. It is possible that smaller mtDNA molecules with a replicative advantage increased in proliferation (Rand, 2001; Phillips et al., 2015). The AT-region is highly repetitive and problematic to sequence, making this hypothesis difficult to evaluate and future experimental is needed to investigate these possibilities.

### Indel mutations

A previous analysis found homopolymers to be hotspots for indel mutation in *C. elegans* mtDNA (Denver et al., 2000); we also observed numerous indel mutations in this study and, applying improved sequencing technology, were able to provide quantitative estimates of heteroplasmic indel frequencies. All N2 derived MA lines and four *gas-1* derived MA lines harbored indel heteroplasmies in the *ATP6* gene sequence, with no evidence for the heteroplasmy in either the N2 or *gas-1* progenitor (Table 4). This result suggests that this specific site may be a hotspot for indel mutation in *C. elegans*. Similarly, as two N2 derived MA lines (MA523 and MA 574) experienced insertion of a “T” allele at site 11721 located in the *NAD-5* gene, and the N2 progenitor was observed to only harbor the wild-type allele at this position, the N2 strain may have predisposition for insertion events at this site. We cannot, however, rule out that these indels may have been segregating at extremely low (undetectable) frequencies in the N2 ancestor.

The fact that we observed more indel variants in N2 derived lines compared to *gas-1* nematodes is consistent with their much larger number of MA generations. However, the higher mtDNA copy number in N2 vs. *gas-1* MA lines may also contribute to this finding. Studies in mice have determined elevated mtDNA copy number to be associated with impaired replication, which may result in mtDNA deletions (Fan et al., 2008).

### SNP mutations

Our study found more mtDNA base substitution events among the *gas-1* G43 MA lines compared to the N2 G250 MA lines (Figure 2). The larger number of heteroplasmic SNPs observed in the *gas-1* derived lines may be a product of impaired mitochondrial activity and/or high ROS levels reported as phenotypes for the *gas-1* mutant (Pujol et al., 2013). However, our numbers of detected SNPs were small (only two SNPs in N2 lines and seven in *gas-1* lines), and further work will be required to establish whether any relationship exists between mitochondrial ETC dysfunction and mtDNA mutation. We preferred to take a conservative approach to identifying putative heteroplasmies because our coverage was not extraordinarily high (mean mtDNA coverage = 545X, Table 1). We recognize that our analysis likely results in false negatives although this approach minimizes the possibilities of false positives.

A previous study of mtDNA mutations in *C. elegans* N2 MA lines found homopolymer runs to be mutation hotspots, although there was no evidence for putative base-substitution hotspot sites (e.g., parallel mutation) (Denver, 2000). Here, two *gas-1* MA lines (MA431 and MA438) experienced the same heteroplasmic mutation at 211 bp located in the *NAD-6* gene. As this mutation was absent from both wild-type and *gas-1* progenitor, as well as all N2-derived MA lines, this result is consistent with this site being a mutation hotspot.

We observed a low-frequency heteroplasmy in the *gas-1* progenitor at position 8439 bp in the *COX-1* gene. The same heteroplasmy was observed to fix in one line (MA438) and achieve a relatively high frequency (>37%) in another (MA412) (Table 4). Results of Sanger sequencing revealed that this heteroplasmy was maintained at various levels among individual nematodes sampled from the *gas-*1 progenitor, including complete absence (non-detectable) in some individuals and fixation in others. Furthermore, one explanation for the rapid shift in mitochondrial genotypes observed trans-generationally is a mitochondrial genetic “bottleneck” (high genetic drift) where only a small proportion of mitochondrial genomes repopulate the subsequent generation (Chinnery et al., 2000). Our results may be in alignment with predictions of these theories where only a small subset of mtDNA molecules from the parental generation repopulate the mitochondrial genome pool for the progeny. However, an important caveat should be noted: the studies referenced here used mammalian models and were not conducted on *C. elegans*. It still unknown how many mtDNA molecules from the mother are used to proliferate the population of mtDNA in germ cells in the *C. elegans* model.

Eight out of 18 individual *gas-1* progenitor worms contained the *COX-1* 8439 heteroplasmy. This observation highlights the reality that there exists variation among individuals in the progenitor lab population, and that subsequent observations in lab-evolved lines will be affected by the particular nematode randomly chosen to initiate each plate. Moreover, three of the 14 *gas-1* derived MA lines analyzed by Sanger sequencing were found to harbor homoplasmic genomes fixed for the 8439 bp mutation. In light of both these observations, it is highly likely that the *gas-1* progenitor employed to generate the *gas-1* MA lines harbored the *COX-1* heteroplasmy at moderately high levels. Our results indicate that mtDNA heteroplasmies can become fixed in individuals from lineages evolving under minimized natural selection.

## Conclusion

This study provides new insights into the heteroplasmic paths of mutation in an animal mitochondrial genome. Our study of mtDNA variation across experimental generations revealed increases in mtDNA copy number in *C. elegans* wildtype and *gas-1* strains across generations under extreme genetic drift, adding to previous studies demonstrating increased mtDNA copy number across nematode development (Bratic et al., 2009). The higher frequency of detected single-base substitution mutations in *gas-1* MA-line genomes relative to that of indels may suggest increased vulnerability of the *gas-1* genotype to experience single-base substitution events relative to wild-type MA lines. It is, however, unclear whether the differences in mtDNA mutation type observed between the two sets of MA lines are a consequence of differences in underlying rates of mutation to each type and/or of subsequent evolutionary dynamics. Further insights into the heteroplasmic mtDNA mutation process will benefit from approaches that consider many generational timepoints, a variety of starting genotypes, and more careful examination of the specific progenitor individuals used to initiate experimental evolution.

## Author contributions

RW led the research, led the analysis, wrote the manuscript, and contributed to experimental design. DH contributed to research and contributed to manuscript writing. DD, SE contributed to manuscript writing and led the experimental design.

## Funding

This research was supported by NIH grant GM087628 and NSF grant MCB-1330427.

### Conflict of interest statement

The authors declare that the research was conducted in the absence of any commercial or financial relationships that could be construed as a potential conflict of interest.
