# Supplementary material for: Paths of Heritable Mitochondrial DNA Mutation and Heteroplasmy in Reference and gas-1 Strains of Caenorhabditis elegans
Source: Front Genet. 2016 Apr 13;7:51. doi: 10.3389/fgene.2016.00051 (PMC4829587; doi:10.3389/fgene.2016.00051)
Supplement: Supplementary file 1 [file DataSheet1.pdf]

Supplementary Table 1: Number of Generations of Bottlenecking Per Line

**Line**	**Number Generations Bottlenecking**
**N2 Line**	
Progenitor	N/A
N2 MA523	250
N2 MA526	250
N2 MA529	250
N2 MA553	250
N2 MA574	250
***gas-1* Line**	
progenitor	N/A
gas-1 MA412	42
gas-1 419	38
gas-1 MA429	43
gas-1 MA431	44
gas-1 MA438	43

All lines experienced single-worm bottlenecking. Single worms transferred in 4 day intervals. All nematodes maintained on standard NGM plates.

Supplementary Table 2: Kruskal Wallis H Test of mtDNA copy number

	DF	Chi-Sq	P-Value
Strain	11	154.2	2.2×10^{-16}

Kruskal Wallis H Test analysis of mtDNA copy number with strain as an explanatory variable. Degrees of freedom (DF) and Chi-Squared value (Chi-Sq).

Supplementary Table 3: Kruskal Multiple Comparison test of mtDNA copy number

**Line 1**	**Line 2**	**obs.dif**	**critical.dif**	**difference**
N2 Progenitor	gas-1 Progenitor	6.71429	61.91712	FALSE
N2 Progenitor	MA412	37.9286	61.91712	FALSE
N2 Progenitor	MA419	72.2857	61.91712	TRUE
N2 Progenitor	MA429	80.2143	61.91712	TRUE
N2 Progenitor	MA431	43.0714	61.91712	FALSE
N2 Progenitor	MA438	30.8571	61.91712	FALSE
N2 Progenitor	MA523	45.5	61.91712	FALSE
N2 Progenitor	MA526	142.5	61.91712	TRUE
N2 Progenitor	MA529	103.714	61.91712	TRUE

N2 Progenitor	MA553	121.5	61.91712	TRUE
N2 Progenitor	MA574	119.429	61.91712	TRUE
gas-1 Progenitor	MA412	44.6429	61.91712	FALSE
gas-1 Progenitor	MA419	79	61.91712	TRUE
gas-1 Progenitor	MA429	86.9286	61.91712	TRUE
gas-1 Progenitor	MA431	49.7857	61.91712	FALSE
gas-1 Progenitor	MA438	37.5714	61.91712	FALSE
gas-1 Progenitor	MA523	52.2143	61.91712	FALSE
gas-1 Progenitor	MA526	149.214	61.91712	TRUE
gas-1 Progenitor	MA529	110.429	61.91712	TRUE
gas-1 Progenitor	MA553	128.214	61.91712	TRUE
gas-1 Progenitor	MA574	126.143	61.91712	TRUE
MA412	MA419	34.3571	61.91712	FALSE
MA412	MA429	42.2857	61.91712	FALSE
MA412	MA431	5.14286	61.91712	FALSE
MA412	MA438	7.07143	61.91712	FALSE
MA412	MA523	7.57143	61.91712	FALSE
MA412	MA526	104.571	61.91712	TRUE
MA412	MA529	65.7857	61.91712	TRUE
MA412	MA553	83.5714	61.91712	TRUE
MA412	MA574	81.5	61.91712	TRUE
MA419	MA429	7.92857	61.91712	FALSE
MA419	MA431	29.2143	61.91712	FALSE
MA419	MA438	41.4286	61.91712	FALSE
MA419	MA523	26.7857	61.91712	FALSE
MA419	MA526	70.2143	61.91712	TRUE
MA419	MA529	31.4286	61.91712	FALSE
MA419	MA553	49.2143	61.91712	FALSE
MA419	MA574	47.1429	61.91712	FALSE
MA429	MA431	37.1429	61.91712	FALSE
MA429	MA438	49.3571	61.91712	FALSE
MA429	MA523	34.7143	61.91712	FALSE
MA429	MA526	62.2857	61.91712	TRUE
MA429	MA529	23.5	61.91712	FALSE
MA429	MA553	41.2857	61.91712	FALSE

MA429	MA574	39.2143	61.91712	FALSE
MA431	MA438	12.2143	61.91712	FALSE
MA431	MA523	2.42857	61.91712	FALSE
MA431	MA526	99.4286	61.91712	TRUE
MA431	MA529	60.6429	61.91712	FALSE
MA431	MA553	78.4286	61.91712	TRUE
MA431	MA574	76.3571	61.91712	TRUE
MA438	MA523	14.6429	61.91712	FALSE
MA438	MA526	111.643	61.91712	TRUE
MA438	MA529	72.8571	61.91712	TRUE
MA438	MA553	90.6429	61.91712	TRUE
MA438	MA574	88.5714	61.91712	TRUE
MA523	MA526	97	61.91712	TRUE
MA523	MA529	58.2143	61.91712	FALSE
MA523	MA553	76	61.91712	TRUE
MA523	MA574	73.9286	61.91712	TRUE
MA526	MA529	38.7857	61.91712	FALSE
MA526	MA553	21	61.91712	FALSE
MA526	MA574	23.0714	61.91712	FALSE
MA529	MA553	17.7857	61.91712	FALSE
MA529	MA574	15.7143	61.91712	FALSE
MA553	MA574	2.07143	61.91712	FALSE

All possible pairwise comparisons of mtDNA copy number between lines. Critical Difference (critical.dif) signifies level delineating significance from non-significance. Observational difference (obs.dif) indicates observed difference between lines. P <0.05 indicates significance of mtDNA copy number difference between two lines indicated by corresponding Output (difference). TRUE signifies the two lines are significantly different in mtDNA copy number, FALSE indicates the two lines are not significantly different from one another.

Supplementary Table 4: Sanger Results for Mitochondrial Position 8439 Heteroplasmy of Individual *gas-1* progenitor L1 Worms

***gas-1* G0 L1 Sample**	**Reference Base**	**Evidence "C" Allele**	**Evidence "A" Allele**	**Phred Score**	**Average Flanking Phred Score**
1	C	Heteroplasmy	Heteroplasmy	46	60.02
2	C	Heteroplasmy	Heteroplasmy	12	57.44
3	C	Heteroplasmy	Heteroplasmy	25	59.2
4	C	Heteroplasmy	Heteroplasmy	43	58.7
5	C	Fixed	0	59	59.2
6	C	Heteroplasmy	Heteroplasmy	27	60.44
7	C	Fixed	0	59	57.57
8	C	Fixed	0	62	61.41
9	C	Fixed	0	62	59.23
10	C	Heteroplasmy	Heteroplasmy	34	58.58
11	C	Fixed	0	62	59.68
12	C	Heteroplasmy	Heteroplasmy	18	59.1
13	C	Fixed	0	36	41.07
14	C	Heteroplasmy	Heteroplasmy	34	54.99
15	C	Fixed	0	54	55.82
16	C	Fixed	0	56	40.45
17	C	Fixed	0	59	56.81
18	C	Fixed	0	54	58.7

All nematodes at L1 stage. Average flanking Phred score calculated by mean Phred score of 50 bp flanking heteroplasmy in both directions.

Supplementary Table 5: Sanger Results for Mitochondrial Position 8439 Heteroplasmy of Individual *gas-1* MA412 L1 Worms

***gas-1* MA 412 L1 Sample**	**8439 Allele Call**	**Evidence "C" Allele**	**Evidence "A" Allele**	**Phred Score**	**Average Flanking Phred Score**
1	A	Heteroplasmy	Heteroplasmy	23	59.21
2	C	Heteroplasmy	Heteroplasmy	13	58.15
3	C	Heteroplasmy	Heteroplasmy	34	60.14
4	C	Heteroplasmy	Heteroplasmy	18	59.4
5	A	Heteroplasmy	Heteroplasmy	13	59.6
6	A	Heteroplasmy	Heteroplasmy	18	58.26
7	A	Heteroplasmy	Heteroplasmy	23	59.24
8	C	Heteroplasmy	Heteroplasmy	38	59.53
9	C	Fixed	0	62	60.14
10	C	Heteroplasmy	Heteroplasmy	18	58.63
11	C	Heteroplasmy	Heteroplasmy	35	58.78
12	C	Heteroplasmy	Heteroplasmy	28	58.29
13	C	Heteroplasmy	Heteroplasmy	13	58.75
14	C	Heteroplasmy	Heteroplasmy	27	58.6
15	C	Fixed	0	59	58.98
16	C	Heteroplasmy	Heteroplasmy	18	53.45
17	A	Heteroplasmy	Heteroplasmy	13	50.16

All nematodes at L1 stage. Average flanking Phred score calculated by mean Phred score of 50 bp flanking heteroplasmy in both directions.

Supplementary Table 6: Sanger Results for Mitochondrial Position 8439 Heteroplasmy of Individual *gas-1* MA429 L1 Worms

***gas-1* MA 429 L1 Sample**	**8439 Allele Call**	**Evidence "C" Allele**	**Evidence "A" Allele**	**Phred Score**	**Average Flanking Phred Score**
1	C	Fixed	0	62	61.19
2	C	Fixed	0	62	60.41
3	C	Fixed	0	62	60.97
4	C	Fixed	0	62	60.63
5	C	Fixed	0	62	60.84
6	C	Fixed	0	62	60.81
7	C	Fixed	0	62	60.46
8	C	Fixed	0	62	60.99
9	C	Fixed	0	62	60.76
10	C	Fixed	0	62	59.74
11	C	Fixed	0	62	60.02
12	C	Fixed	0	62	60.26
13	C	Fixed	0	62	60.65
14	C	Fixed	0	62	60.46
15	C	Fixed	0	56	60.14
16	C	Fixed	0	62	60.86
17	C	Fixed	0	62	61
18	C	Fixed	0	54	51.75

All nematodes at L1 stage. Average flanking Phred score calculated by mean Phred score of 50 bp flaning heteroplasmy in both directions.

Supplementary Table 7: Sanger Results for Mitochondrial Position 8439 Heteroplasmy of Individual *gas-1* MA438 L1 Worms

***gas-1* MA 438 L1 Sample**	**8439 Allele Call**	**Evidence "C" Allele**	**Evidence "A" Allele**	**Phred Score**	**Average Flanking Phred Score**
1	A	0	Fixed	62	61.53
2	A	0	Fixed	62	60.85
3	A	0	Fixed	62	60.77
4	A	0	Fixed	62	61.33
5	A	0	Fixed	62	61.2
6	A	0	Fixed	62	61.64
7	A	0	Fixed	62	61.21
8	A	0	Fixed	62	56.56
9	A	0	Fixed	62	59.98
10	A	0	Fixed	62	60.44
11	A	0	Fixed	62	60.48
12	A	0	Fixed	62	59.66
13	A	0	Fixed	62	60.24
14	A	0	Fixed	62	60.73
15	A	0	Fixed	56	58
16	A	0	Fixed	62	60.53
17	A	0	Fixed	62	56.59

All nematodes at L1 stage. Average flanking Phred score calculated by mean Phred score of 50 bp flanking heteroplasmy in both directions.

Supplementary Table 8: Sanger Results for Mitochondrial Position 8439 Heteroplasmy of Bulk *gas-1* and N2 Progenitor Worms

**Progenitor Line**	**8439 Allele**	**Evidence "C" Allele**	**Evidence "A" Allele**	**Phred Score**	**Average Flanking Score**
N2	C	Fixed	0	62	61.62
gas-1	C	Fixed	0	62	61.53

All nematodes at L1 stage. Average flanking Phred score calculated by mean Phred score of 50 bp flanking heteroplasmy in both directions.

Supplementary Table 9: Sanger Results for Mitochondrial Position 8439 Heteroplasmy of Bulk Extraction *gas-1* MA lines

***gas-1* MA Sample**	**Reference Base**	**Evidence "C" Allele**	**Evidence "A" Allele**	**Phred Score**	**Average Flanking Phred Score**
MA402	C	Fixed	0	62	61.76
MA405	C	Fixed	0	62	61.67
MA412	C	Heteroplasmy	Heteroplasmy	18	58.37
MA415	C	Fixed	0	62	59.59
MA422	C	Fixed	0	62	61.18
MA425	A	Heteroplasmy	Heteroplasmy	18	55.83
MA427	C	Fixed	0	62	61
MA428	C	Fixed	0	62	60.98
MA429	C	Fixed	0	62	58.95
MA432	C	Fixed	0	56	61.01
MA433	C	Fixed	0	62	60.82
MA437	A	0	Fixed	62	61.41
MA438	A	0	Fixed	62	60.18
MA439	A	0	Fixed	62	61.41

All nematodes at L1 stage. Average flanking Phred score calculated by mean Phred score of 50 bp flanking heteroplasmy in both directions.

Supplementary Figure 1: Box and whisker plot of Normalized mtDNA copy number per line

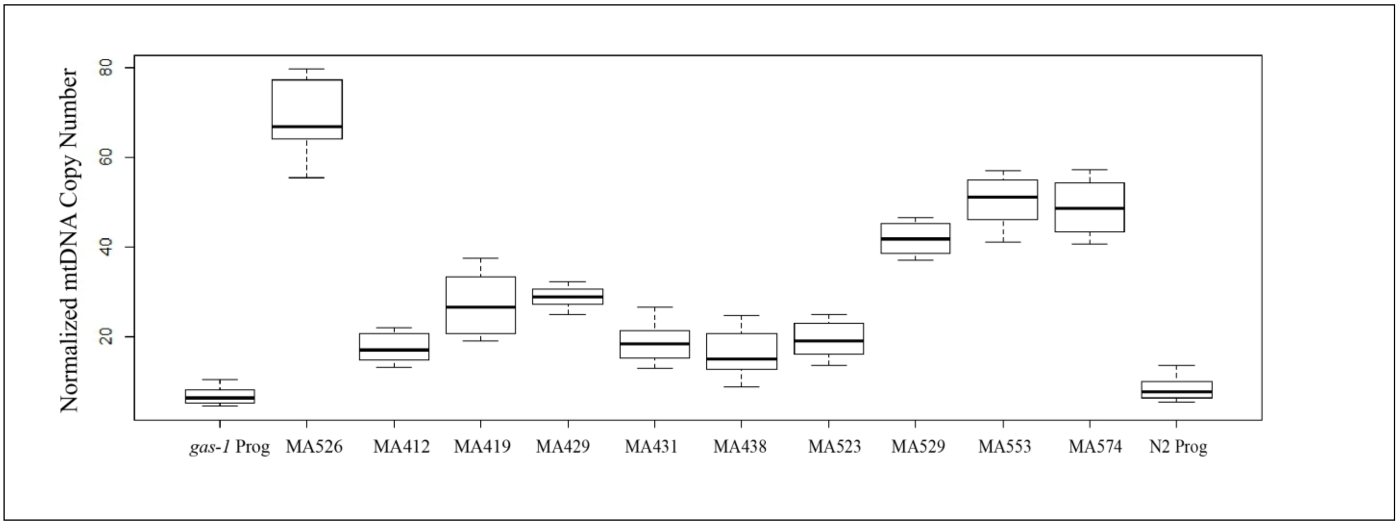

Plot depicting variance for normalized mtDNA copy number in all 12 lines. Normalized mtDNA copy number portrayed on Y-axis.
